# Coding Mutations in Vacuolar Protein-Sorting 4 AAA+ ATPase Endosomal Sorting Complexes Required for Transport Protein Homologs Underlie *bc-2* and New *bc-4* Gene Conferring Resistance to *Bean Common Mosaic Virus* in Common Bean

**DOI:** 10.3389/fpls.2021.769247

**Published:** 2021-12-13

**Authors:** Alvaro Soler-Garzón, Phillip E. McClean, Phillip N. Miklas

**Affiliations:** ^1^Irrigated Agriculture Research and Extension Center, Washington State University, Prosser, WA, United States; ^2^Department of Plant Sciences, North Dakota State University, Fargo, ND, United States; ^3^Grain Legume Genetics and Physiology Research Unit, USDA-ARS, Prosser, WA, United States

**Keywords:** genome-wide association study, gene homology, host-pathogen interaction, *Phaseolus vulgaris*, potyvirus, marker-assisted selection

## Abstract

*Bean common mosaic virus* (BCMV) is a major disease in common bean (*Phaseolus vulgaris* L.). Host plant resistance is the most effective strategy to minimize crop damage against BCMV and the related *Bean common mosaic necrosis virus* (BCMNV). To facilitate breeding for resistance, we sought to identify candidate genes and develop markers for the *bc-2* gene and the unknown gene with which it interacts. Genome-wide association study (GWAS) of the Durango Diversity Panel (DDP) identified a peak region for *bc-2* on chromosome Pv11. Haplotype mapping narrowed the *bc-2* genomic interval and identified Phvul.011G092700, a vacuolar protein-sorting 4 (Vps4) AAA+ ATPase endosomal sorting complexes required for transport (ESCRT) protein, as the *bc-2* candidate gene. The race Durango Phvul.011G092700 gene model, *bc-2*^[UI^ ^111]^, contains a 10-kb deletion, while the race Mesoamerican *bc-2*^[Robust]^ consists of a single nucleotide polymorphism (SNP) deletion. Each mutation introduces a premature stop codon, and they exhibit the same interaction with the pathogroups (PGs) tested. Phvul.005G125100, another Vps4 AAA+ ATPase ESCRT protein, was identified as the candidate gene for the new recessive *bc-4* gene, and the recessive allele is likely an amino acid substitution in the microtubule interacting and transport (MIT) domain. The two Vps4 AAA+ ATPase ESCRT proteins exhibit high similarity to the *Zym* Cucsa.385040 candidate gene associated with recessive resistance to *Zucchini yellow mosaic virus* in cucumber. *bc-2* alone has no resistance effect but, when combined with *bc-4*, provides resistance to BCMV (except PG-V) but not BCMNV, and, when combined with *bc-u*^d^, provides resistance to BCMV (except BCMV PG-VII) and BCMNV. So instead of different resistance alleles (i.e., *bc-2* and *bc-2*^2^), there is only *bc-2* with a differential reaction based on whether it is combined with *bc-4* or *bc-u^d^*, which are tightly linked in repulsion. The new tools and enhanced understanding of this host-virus pathogen interaction will facilitate breeding common beans for resistance to BCMV and BCMNV.

## Introduction

*Bean common mosaic virus* (BCMV) and *Bean common mosaic necrosis virus* (BCMNV) are closely related positive-stranded RNA potyviruses that limit common bean (*Phaseolus vulgaris* L.) production worldwide. BCMV and BCMNV are transmitted by aphids, mechanically, or through infected seeds. Genetic resistance is the most effective means to control BCMV and BCMNV. For a comprehensive review of these viruses and their interactions with the common bean, refer to Worrall et al. (2015).

The long-standing host-pathogen interaction model developed by Drijfhout (1978) for these two viruses and common bean was based on six host recessive resistance alleles (i.e., *bc-1* and *bc-1*^2^ alleles, *bc-2* and *bc-2*^2^ alleles, *bc-3*, and *bc-u*) distributed across four loci and the dominant *I* gene. The presence of the *bc-u* “ubiquitous” gene was necessary for the expression of the other recessive resistance genes, in the absence of the *I* gene. Isolate diversity was classified into seven pathogroups (PGs), i.e., PG-I to PG-VII, based on interactions with 11 host groups (HGs), i.e., HG-1 to HG-11, which possessed different combinations of resistance genes. Recent adjustments to the model include as follows: (i) a new PG (PG-VIII) with a BCMV isolate that overcomes *bc-3* gene in the HG-7 genotype IVT-7214 (Feng et al., 2015); (ii) a new HG-12 with *I* and *bc-3* resistance genes (Larsen et al., 2005); (iii) only one resistance allele “*bc-1*” for the *Bc-1* locus (Soler-Garzón et al., 2021); and (iv) absence of the *bc-u* “helper” gene in HG-2, -4, -5, and -7 where presence was expected. Soler-Garzón et al. (2021) used the new symbol “*bc-u*^d^” to designate the presence of *bc-u* in HG-3, -6, -10, and -11. They observed that *bc-u*^d^ may “help” *bc-2*^2^ to condition resistance to BCMV and BCMNV in genotypes assigned to HG-6 and HG-11. However, those interactions need to be validated, and a *bc-u*-like gene for *bc-2* in genotypes assigned to HG-4 and HG-5 has not been identified.

Candidate genes were recently characterized for *bc-1* (i.e., receptor-like protein kinases) on chromosome 3 (Pv03) and *bc-u*^d^ (i.e., basic leucine zipper transcription factor protein) on Pv05 (Soler-Garzón et al., 2021). A eukaryotic translation initiation factor 4E protein (eIF4E) with a reported role in potyviral infection (Naderpour et al., 2010) was identified as the candidate gene for *bc-3* on Pv06. The *I* gene on Pv02, which induces an immune response to BCMV or temperature-dependent hypersensitive response (HR) to select a few BCMV strains as well as temperature-independent HR to BCMNV infection, was associated with a cluster of genes encoding nucleotide-binding site-leucine-rich repeat (NBS-LRR) proteins (Vallejos et al., 2006). The *Bc-2* locus and the unknown gene that interacts with *bc-2* in HG-4 and HG-5 have been neither associated with candidate genes nor mapped yet, limiting the utilization of the *bc-2* and *bc-2*^2^ alleles in breeding for resistance to BCMV and BCMNV.

To facilitate breeding for resistance to BCMV and BCMNV and to gain a better understanding of the host-pathogen model, our objectives were to map, identify candidate genes, and develop markers for *bc-2* and the unknown “helper” gene it interacts with, in HG-4 and HG-5 categorized lines.

## Materials and Methods

### Plant Materials

A total of 182 dry bean lines from the Durango Diversity Panel (DDP), described in a previous study by Soler-Garzón et al. (2021), were used in this study to map and target candidate genes for *bc-2/bc-2*^2^ resistance alleles and the unknown gene that interacts with *bc-2*. The Othello/VAX 3 (OV3) F_6:7_ recombinant inbred line (RIL) population (*n* = 100) (Viteri et al., 2014) was used to narrow the *bc-2*^2^ genomic interval. Othello pinto bean cultivar (NW-410/2/Victor/Aurora; Burke et al., 1995) possesses the *bc-1*/*bc-2*^2^/*bc-u*^d^ resistance genes (Soler-Garzón et al., 2021), which places it in HG-6. Othello is only susceptible to BCMV PG-VII strains such as US-6 and NL-4. VAX 3 is a germplasm release derived from a multiple-parent interspecific cross between common bean and tepary bean (*Phaseolus acutifolius*). VAX 3 has the *I* gene for resistance to BCMV (Singh et al., 2001).

Seven F_2_ populations were generated from crosses among DDP lines selected for presence vs. absence of the *bc-2* allele based on phenotypic response to different PGs, as reported in the literature, or from inoculations conducted by us. Furthermore, two HG-4 navy bean cultivars, namely, Sanilac and Michelite-62 with *bc-2*, were crossed with Poncho (DDP-041), Beryl (DDP055), and Matterhorn (DDP-033), which lack *bc-2* to generate six additional F_2_ populations segregating for the *bc-2* gene originating from race Mesoamerica (Supplementary Table 1).

### Phenotypic Evaluation

The DDP lines were evaluated for reaction to US-6 (PG-VII) BCMV strain (Silbernagel, 1969). DDP reactions to NL-8 (PG-III) and NL-3 (PG-VI) BCMNV strains were reported by Soler-Garzón et al. (2021). The OV3 RILs were evaluated for reaction to US-6, NL-8, and NL-3 strains and the BCMV US-4 (PG-IV) strain (Skotland and Burke, 1961) was used to inoculate a subset of OV3 RILs. The subsets of individual plants from F_2_ populations and F_2:3_ families (i.e., progeny lines) were evaluated for reaction to one or more strains, which included US-4 (PG-IV) and US-6 (PG-V11) BCMV strains as well as NL-8 (PG-III) and NL-3 (PG-VI) BCMNV strains.

All virus strains were maintained in the universal susceptible HG-1 differential cultivar “Sutter Pink,” with the youngest leaves with mosaic symptoms used as inoculum. All inoculations were conducted in the USDA-ARS greenhouses at Prosser, WA, United States, under controlled conditions (22–28°C temperature range and 14-h photoperiod using artificial lights as necessary). Test plants were sown in 9 cm^3^ pots (three plants maximum per pot) containing a commercial potting mix (Sun Gro Horticulture, Bellevue, WA, United States). About 10 days after planting, the two primary leaves of each plant were mechanically inoculated with the same strain using the procedure described by Drijfhout (1978).

Disease reactions were observed every week until 5 weeks post-inoculation (wpi). The symptoms for each plant, as reported by Soler-Garzón et al. (2021), were recorded as follows: NS = no apparent symptoms on inoculated leaves and no systemic symptoms; M = leaf curling and plant stunting with severe systemic chlorotic mosaic symptoms; mM = mild systemic chlorotic mosaic symptoms; dM = delayed severe systemic chlorotic mosaic symptoms observed 2–4 wpi; VN = restricted vein necrosis on inoculated leaves, no systemic symptoms; VN^+^ = restricted vein necrosis on inoculated leaves, with some small patches (10 mm^2^) of systemic restricted vein necrosis on upper trifoliolate leaves observed from 3 to 5 wpi; NLL = local necrotic pinpoint lesions on inoculated leaves, no systemic symptoms; TN = lethal systemic top necrosis by 7–10 days post-inoculation (dpi), resulting in plant death; and dTN = delayed top necrosis beginning >11 dpi, most often resulting in plant death. Host differentials with specific reactions to BCMV and BCMNV strains in parenthesis (US-4/US-6/NL-8/NL-3): HG-1, Sutter pink (M/M/M/M); HG-4, UI-34 (NS/NS/M/M); HG-6, Othello (NS/M/NS/NS); HG-9, Topcrop (NS/NS/VN/TN); HG-10, Beryl (NS/NS/VN/VN); and HG-9b, Jubila (TN/NS/VN/VN^+^) were included as controls.

### DNA Extraction and Genotyping

Genomic DNA was extracted from 20 mg of leaf tissue from an individual plant for each DDP accession, check line, and HG differential cultivar using a QIAGEN DNeasy 96 Plant Kit (Hilden, Germany). For F_2_ and F_3_ plants and single plants of OV3 RILs, DNA was extracted from four-leaf disks (approximately 30 mm^2^) according to the alkaline extraction method described by Xin et al. (2003) with modifications (Soler-Garzón et al., 2021). A 1:7 dilution of the extracted DNA was placed in a 96-well plate with a final volume of 100 μl. Finally, 5 μl of diluted DNA template was used for PCR.

The DDP was genotyped, as reported by Soler-Garzón et al. (2021), with a filtered set of 1,269,044 biallelic single-nucleotide polymorphisms (SNPs) obtained by whole-genome resequencing. The linkage map for OV3, which was generated with polymorphic SNPs from the BARCBean6K_3 BeadChip assay (Song et al., 2015), was obtained from Viteri et al. (2014). The DDP and OV3 RILs were assayed for the *I*-, *bc-1*-, *bc-3*-, and *bc-u*^d^-linked SNP markers described by Soler-Garzón et al. (2021). The individual F_2_ plants from each population were similarly assayed with the same resistance gene-linked markers. Eventually, the abovementioned materials were assayed with the new putative markers for *bc-2* and unknown gene (described below). Notably, 35 select F_2:3_ progenies from individual F_2_ plants, either fixed or heterozygous for one or more of the resistance gene-linked markers, were genotyped using this same set of SNP markers. Eight true-breeding F_2:3_ families were used as controls for each gene in a homozygous state, and the remaining 27 families had at least 1 gene in a heterozygous state (Supplementary Table 1). A total of 217 F_2_ plants and 1,345 F_2:3_ plants were assayed for the *I*, *bc-1*, *bc-3*, *bc-u*^d^, and new *bc-2* and unknown gene, i.e., gene-linked SNP markers (Supplementary Table 2).

### Genome-Wide Association Study Analysis

The phenotypic reactions to US-6, NL-8, and NL-3 strains and the genotypic SNP data for the DDP were integrated for the genome-wide association study using a multi-locus random-SNP-effect mixed linear model (mrMLM) described by Wang et al. (2016) and implemented in the “mrMLM” R package (Wen et al., 2018). A kinship matrix was generated using the efficient mixed-model association (EMMA) algorithm implemented in the Genome Association and Prediction Integrated Tool (GAPIT) R package (Lipka et al., 2012) with corrections for kinship and population structure. Five principal components (PCs) generated from GAPIT were included as covariates. The Bonferroni test was implemented to control the experiment-wise type I error rate at 0.05. The GWAS results were plotted using CMPlot v3.62 (Yin, 2016), and IntAssoPlot v3 (He et al., 2020) was used to represent regional-based marker-trait associations graphically.

### Toward Candidate Gene Markers for *bc-2* and Unknown Gene

Candidate genes located within the identified genomic intervals were obtained by perusing available scientific literature and aligning their sequences to the Andean G19833 v2.1 and Middle-American UI 111 v1.1 *P. vulgaris* reference genomes.^1^ Subsequently, exon sequences of each candidate gene were amplified across select genotypes using PCR primers designed with Primer3 software (Koressaar and Remm, 2007; Untergasser et al., 2012; Supplementary Table 3).

Two standard PCRs, for each sample, were replicated in a volume of 25 μl containing 1.8 mM MgCl_2_, 0.4 mM of deoxynucleoside triphosphate (dNTP) Mix (Promega™, Madison, WI, United States), 0.25 μM of each primer (forward and reverse), 25 ng of genomic DNA, and 1 unit Taq DNA polymerase (Promega), in 1× PCR buffer (Promega), under the following amplification conditions: 2 min at 95°C, followed by 38 cycles at 94°C for 20 s, specific annealing temperature for each primer set for 30 s and 72°C for 90 s, and a final extension at 72°C for 5 min. All amplifications were performed in a PCR Eppendorf Mastercycler (Eppendorf AG, Hamburg, Germany). PCR fragments were visualized by gel electrophoresis on 2% (w + v) agarose and then purified and Sanger sequenced by Eurofins MWG Operon (Louisville, KY, United States). Sequence trimming, alignment, and polymorphism discovery were performed with Geneious 9.1.2 software (Kearse et al., 2012).

### Candidate Single-Nucleotide Polymorphism Marker Verification

For each polymorphic variant identified in coding regions of the sequenced candidate genes, a set of allele-specific primers, with GC tail of unequal length attached to their 5′ end, were designed using the Primer3 software (Koressaar and Remm, 2007; Untergasser et al., 2012), according to melting temperature (Tm)-shift SNP genotyping method developed by Wang et al. (2005). Fragments were amplified by PCR on an Eppendorf Mastercycler using a volume of 20-μl master-mix containing 1.5 mM MgCl_2_, 0.2 mM of dNTP Mix (Promega), 0.15 μM of each primer, 1X EvaGreen™ (Biotium, Fremont, CA, United States), 20 ng of genomic DNA, 1X Taq buffer, and 0.1 μl *Taq*1 polymerase (Promega) under the following profile: 94°C for 2 min, then 38 cycles of denaturation at 92°C for 20 s, annealing for 20 s (i.e., the temperature was specific to each primer trio), extension at 72°C for 20 s, and a final extension at 72°C for 5 min. The melting point analysis for the allele determination of the template DNA was performed with a fluorescence-detecting thermocycler (LightCycler™ 4890 Instrument II, Roche, Basel, Switzerland) with EvaGreen™ Fluorescent Dye (Biotium). The fluorescent detection profile was for 1 min at 95°C, and the melting curve step was ramping from 65 to 95°C in increments of 1°C for every 20 s.

## Results

### Identification of the *bc-2* Locus by Genome-Wide Association Study

**FIGURE 1:**
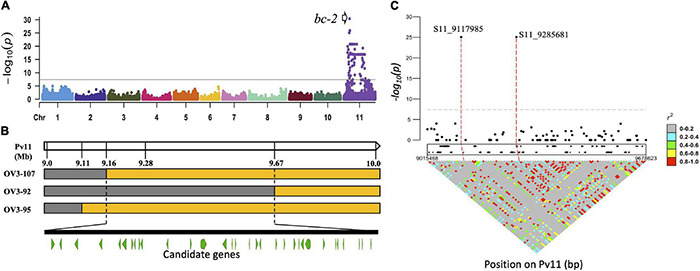
Detection and physical position of *bc-2* gene on Pv11. **(A)** Genome-wide association study (GWAS) between Durango Diversity Panel (DDP) lines with no symptoms when inoculated with either NL-3 or US-6 strains vs. DDP lines exhibiting mosaic to both strains or top necrosis (TN), mild mosaic (mM), or vein necrosis (VN) to NL-3. **(B)** Othello/VAX 3 (OV3) RILs with recombinant haplotypes in the *bc-2* region (orange color = “Othello” segment and gray = VAX-3 segment). **(C)** Marker-trait associations and linkage disequilibrium (LD) plots for the *bc-2* region.

The GWAS was performed with 23 DDP lines with *bc-2* or *bc-2*^2^ alleles [i.e., 9 lines with *bc-2* (NS to US-6 but M against NL-3) and 14 lines with *bc-2*^2^ (12 lines with M against US-6 but NS to NL-3, and 2 lines with NLL to NL-3)] compared to 146 DDP lines absent the *bc-2* and *bc-2*^2^ alleles as indicated by M to both US-6 and NL-3, or mM, TN, or VN to NL-3. One significant peak interval (α = 0.05; *p*-value = 8.53E-26 to 4.33E-31) was detected between 9,117,985 and 10,468,271 based on Pv11 in G19833 v2.1 reference genome (Figure 1A and Supplementary Table 4a).

The interval for *bc-2*^2^ was narrowed from 9.16 to 9.67 Mb in the OV3 RIL population, based on three RILs (i.e., OV3-92 with VN, OV3-107 with TN, and OV3-95 with NLL reactions to NL-3) with recombinant SNP haplotypes (Figure 1B and Supplementary Table 5). The narrowed region for *bc-2*^2^, when applied to the whole-genome resequencing data for the DDP, revealed a low level of linkage disequilibrium (LD) between 74 single-nucleotide variants (SNVs), 31 insertions/deletions (InDel), and 11 short tandem repeats (STRs) polymorphic variants, distributed across 32 gene models (based on the G19833 v2.1 reference genome) (Figure 1C and Supplementary Table 6).

**FIGURE 2:**
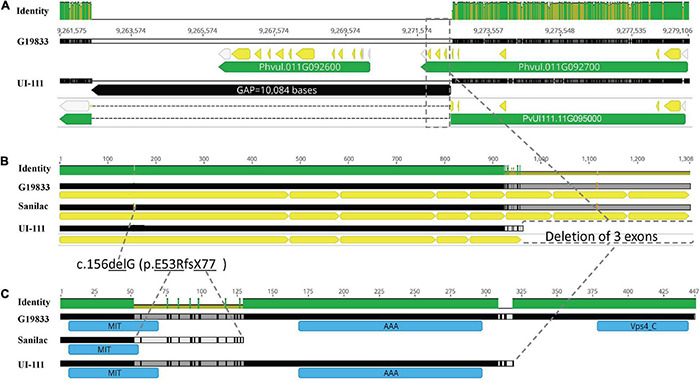
Genomic and protein representation of the candidate gene (*Phvul.011G092700* gene in G19833 v2.1 is synonymous with PvUI111.11G095000 gene in UI 111 v1) for *bc-2*. **(A)** Candidate gene comparison between G19833 and UI 111 reference genomes depicting a deletion in the 3′ region of the candidate gene PvUI111.11G095000 (perpendicular rectangular box outlined by dashed line). **(B)** The Coding Sequence (CDS) of Phvul.011G092700 gene model in a reverse alignment of G19833 (wild-type *Bc-2*), Sanilac (*bc-2*^[*Robust*]^), and UI 111 (*bc-2*^[*UI* 111]^) genotypes. **(C)** Protein prediction *in silico* of Phvul.011G092700 in G19833, Sanilac, and UI 111.

Additional alignment of the *bc-2* region between G19833 v2.1 and UI 111 v1.1 reference genomes revealed a gap of 10,084 bases in “UI 111,” which just so happens to possess the *bc-2* allele based on NS reaction to US-6 strain herein and from published reports (Singh et al., 2007, pp. 987). This 10-kb deletion in “UI 111” spans the Phvul.011G092600 and Phvul.011G092700 gene models of G19833 v2.1 reference genome (Figure 2A). The Phvul.011G092600 model, a subtilisin-like serine protein with endopeptidase activity, is completely missing, and the 3′ end of Phvul.011G092700, a vacuolar protein-sorting 4 (Vps4) AAA+ ATPase endosomal sorting complexes required for transport (ESCRT) protein involved in multivesicular endosome function, is truncated (Figures 2B,C). Amano et al. (2013) identified a Vps4 AAA+ ATPase ESCRT protein as a candidate gene (Cucsa.385040 in Phytozome v13) for the recessive *zym*^A192– 18^ gene conferring resistance to *Zucchini yellow mosaic virus* (ZYMV) in cucumber (*Cucumis sativus* L.). Using BLASTp, the Vps4 AAA+ ATPase ESCRT protein in cucumber had 92.2% identity with the Phvul.011G092700 gene model. Based on functional similarity to *zym*^A192– 18^, Phvul.011G092700 was chosen as the most likely candidate gene for *bc-2*.

### Identification of the “Unknown” Gene (*bc-4*) Revealed by Genome-Wide Association Study and BLASTp

**FIGURE 3:**
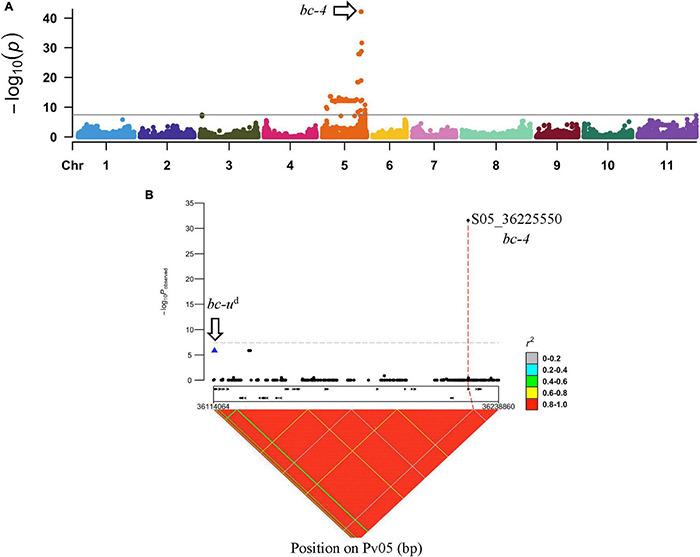
GWAS between DDP lines with no symptoms to US-6 strain vs. DDP lines exhibiting mosaic (M), TN, mM, or VN against NL-3. **(A)** Manhattan plot from GWAS in DDP lines exhibiting a significant peak on Pv05. **(B)** *bc-4* regional association plot showing linkage (in repulsion) between *bc-u*^d^ and *bc-4* (S05_36225550).

To identify the unknown gene that interacts with *bc-2*, GWAS was conducted with 9 DDP lines with *bc-2* as detected by NS to US-6 and M to NL-3 strain compared to 160 DDP lines without *bc-2* as determined by M, mM, NS, TN, and VN reactions against NL-3 strain. Notably, DDP lines without *bc-2* included 12 lines with NS and 2 lines with NLL to NL-3 strain due to the presence of *bc-2*^2^. The Manhattan plot revealed one significant peak (α = 0.05; *p*-value = 6.74E-43 to 2.62E-32) on Pv05, with an interval between 35,471,758 and 36,225,550 bases (Figure 3A and Supplementary Table 4b), which is putatively associated with the “unknown” gene that assists the expression of *bc-2*.

The Vps4 AAA+ ATPase ESCRT protein of the *Phvul.011G092700* candidate gene for the *bc-2* resistance gene was blasted against the G19833 v2.1 reference genome, and the most significant hit (i.e., 94.9% identity) was Phvul.005G125100, a Vps4 AAA+ ATPase ESCRT homolog. Phvul.005G125100 was within the 35,471,758- to 36,225,550-bp GWAS interval. A significant SNP S05_36225550 (*p*-value = 2.62E-32), a missense variant, from the GWAS analysis was located within Phvul.005G125100. Furthermore, SNP S05_36225550 was present in all nine DDP lines with the unknown gene and absent in all DDP lines without the unknown gene. Interestingly, among DDP lines, the SNP S05_36225550 was found to be 111,034 bases upstream and in high LD with the *bc-u^d^* gene that interacts with *bc-1* to confer resistance to BCMNV (Soler-Garzón et al., 2021; Figure 3B). Given these preliminary results, this new gene was assigned the preliminary gene symbol *bc-4*.

### Sequencing the Candidate Genes for *bc-2* and *bc-4*

A total of 16 lines were chosen according to BCMV and BCMNV reactions, gene combinations, and origin, for the exon sequencing of the *bc-2* candidate gene *Phvul.011G092700* and the *bc-4* candidate gene *Phvul.005G125100*.

The Phvul.011G092700 sequences were blasted to the G19833 v2.1 reference genome (which has the *Bc-2* wild-type genotype), revealing eight exon regions (Table 1 and Figure 2A). The Phvul.011G092700 sequences in the race Durango genotypes with either *bc-2* (i.e., UI-111 and UI129) or *bc-2*^2^ (i.e., Othello and 92US-1006) recessive resistance alleles all possessed the same 10,084-bp deletion that truncates three exons from the 3′ end (Figure 2B), providing initial evidence of a single resistance allele. For the race Mesoamerican genotypes Robust, Michelite-62, and Sanilac, also with the *bc-2* resistance allele, there was a single deletion-frameshift mutation (Pv11: 9,278,764 bases) in the first exon of Phvul.011G092700. To track these different *bc-2* mutations, they were assigned a superscript in brackets: *bc-2*^[UI^ ^111]^ for the large 10-kb deletion of three exons found in race Durango lines, and *bc-2*^[Robust]^ for the deletion-frameshift found in navy beans.

**TABLE 1:** Polymorphisms detected within the exon regions for the candidate genes *Phvul.011G092700* and *Phvul.005G125100* for *bc-2* and *bc-4*, respectively, among 16 genotypes.

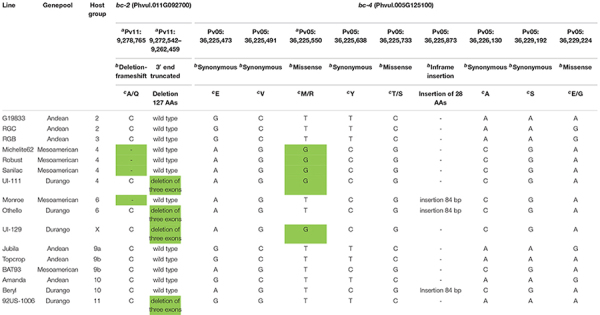

*^a^Candidate variant for marker-assisted selection, with mutant allele shaded light green.*

*^b^Variant type.*

*^c^Amino acid substitutions in protein-coding regions.*

The G19833 Phvul.011G092700 protein is 446 amino acids (aa) in length and contains three domains, namely, (i) microtubule interacting and transport (MIT) domain, (ii) AAA-type ATPase (AAA+) domain, and (iii) Vps4 domain (Figure 2C). The same protein homolog PvUI111.11G095000 (but with a different code) in reference genome “UI 111” (which has *bc-2* genotype) has only 319 aa and 2 domains, i.e., MIT and AAA+, due to the 10,084-bp deletion. The Sanilac (or Robust) homolog has only 128 aa due to 1 deletion-frameshift mutation generating a single aa replacement in aa position 53 (p.E53R) and a premature stop codon in position 77 (fsX77), resulting in an incomplete protein with only the MIT domain.

**FIGURE 4:**
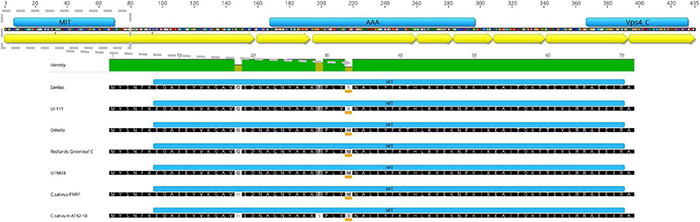
*Phvul.005G125100* candidate gene for *bc-4*. **(Top)** Phvul.005G125100 shows three domains (blue bars) and eight exons (yellow arrows). **(Bottom)** Microtubule interacting and transport (MIT) domain (blue bar) zoomed in for five *Phaseolus vulgaris* lines and two *Cucumis sativus* lines (Amano et al., 2013), depicting the amino acid substitution for position 33 underlined by an orange-colored bar.

The *in silico*-translated Vps4 AAA+ ATPase ESCRT protein of sequenced open reading frame (ORF) for *bc-4* candidate gene *Phvul.005G125100* did not reveal disruption among the MIT, AAA+, and Vps4 domains. The SNP S05_36225550 missense variant was found to be a transversion T > G, which causes the substitution of a non-polar, neutral aa, Methionine (M), with a basic polar, positively charged aa, Arginine (R), at position 33 of the MIT domain (i.e., p.M33R) (Table 1 and Figure 4).

### Tm-Shift Genotyping With Candidate Single-Nucleotide Polymorphism Markers

Tm-shift assays were developed to detect the polymorphisms in the *bc-2* and *bc-4* candidate genes (Table 2). Two distinct assays were designed for the different *bc-2* candidate gene mutations. The *bc-2*^[UI^ ^111]^ assay was developed using the first forward primer aligned to the “UI 111” gene (*PvUI111.11G095000*) sequence and the second forward primer aligned to the G19833 gene (*Phvul.011G092700*) sequence and a common reverse primer (Supplementary Material 1). The technical name for this marker assay is “Pvvps4_del.” For better amplification efficiency, no GC tails were added to “Pvvps4_del” forward primers. The assay for *bc-2*^[Robust]^ flanking the S11_9278764 in-frame-deletion in the navy beans Sanilac and Michelite-62 is named “Pvmit-2_C_del.”

**TABLE 2:** Primers used to generate markers for *bc-2*^[*UI*^ ^111]^, *bc-2*^[*Robust*]^, and *bc-4* alleles.

BCMV resistance alleles	ID marker		Sequence	Ta (°C)	Chr.	Position variant (G19833v2.1)	Sense	Allele resistant	Allele susceptible
*bc-2*^[UI^ ^111]^	Pvvps4_del	Fa	AGACCGTTTGCTAGGTTCACAA	66	Pv11	9,272,542–9,262,459	+	deletion of 10 kb (3 exons)	Wild Type
		R	TGTAGGCAATAAGGCGACGTTT						
		Fb	AAATTATAAACATGTGTTGGCGAGC						
*bc-2* ^[Robust]^	Pvmit-2_C_del	Fa	gcgggcagggcggcATTTCTGCGTGATTGCCTCT	55	Pv11	9,278,765	+	del-C	C
		R	CTTCAAAACGCACCTCAAGTATGA						
		Fb	gcgggcTCTGCGTGATTGCCTCC						
*bc-4*	Pvmit-1_T_G	Fa	gcgggcCGAAAGCGTTCCCTCTCTACAT	70	Pv05	36,225,550	+	G	T
		R	GCGTGATGGCTTCCTTGATCTT						
		Fb	gcgggcagggcggcCGAAAGCGTTCCCTCTCTACAG						

The assay for *bc-4* was developed using the S05_36225550, i.e., missense SNP, in the *Phvul.005G125100* candidate gene. The technical name for this marker is “Pvmit-1_T_G.” These *bc-2* and *bc-4* markers were added to *I*, *bc-1*, *bc-3*, and *bc-u*^d^ markers developed by Soler-Garzón et al. (2021) for genotyping the lines and populations described below.

### Durango Diversity Panel and Othello/VAX 3 Population Assays

The DDP was assayed for both “Pvmit-2_C_del” and “Pvvps4_del” markers for *bc-2*, but only the latter *bc-2*^[UI^ ^111]^-linked marker was detected. All DDP lines with *bc-2* (NS to US-6 and M to NL-3) or *bc-2*^2^ resistance alleles (M to US-6 and NS to NL-3) possessed the “Pvvps4_del” marker for *bc-2*^[UI^ ^11]^, and all other DDP lines possessed the wild-type *Bc-2* allele. The DDP lines with *bc-2* possessed the *bc-4* marker, and those with *bc-2*^2^ possessed the *bc-u*^d^ marker, including those DDP lines in HG-11 (92US-1006 and Quincy) with NLL reaction to NL-3 conditioned by *I*, *bc-2*^2^, and *bc-u*^d^ genes (Supplementary Table 7). These results further support that only one resistance allele, i.e., *bc-2*, exists at the *Bc-2* locus. This *bc-2* allele exhibits a differential effect based on the presence of *bc-4* or *bc-u*^d^.

Four genes (i.e., *I*, *bc-1*, *bc-2*, and *bc-u*^d^) were segregated in the OV3 RIL population. Of the four genes, only the *I* gene had distorted (*df* = 1.0, *p* ≤ 0.05: *X*^2^ = 5.99) segregation, 3:1, in favor of the dominant *I* gene, whereas 1:1 segregation was expected. Altogether, there were six distinct phenotypes to NL-3, seven phenotypes to NL-8, two phenotypes to US-6, and 16 distinct genotypes (Supplementary Table 8). The phenotypic reactions of all RILs to US-6, NL-8, and NL-3 strains matched the genotypes predicted by the resistance gene-linked markers. The most relevant results for this study were that RILs with *bc-2* and *bc-u*^d^ expressed M to US-6 and NS to NL-3 when *I* gene was absent (∼HG-6) and NLL when *I* gene was present (∼HG-11), whereas the *bc-2* allele had no discernible effect against any of the strains in the absence of *bc-u*^d^. Additionally, OV3 RILs with unique genotypes were phenotyped with US-4 (PG-IV) to further validate their use as additional differential cultivars in HGs 1, 2, 6, 8, and 9b (Table 3).

**TABLE 3:** Single-nucleotide polymorphism (SNP) genotyping of host differential genotypes, including OV RILs with new unrepresented genotypes within specific host groups, with reactions to select *Bean common mosaic virus* (BCMV) and *Bean common mosaic necrosis virus* (BCMNV) strains.

		BCMV		BCMNV			*^c^*S02_ 48908259	*^c^*S03_ 4203361	*^b^*Pvvps4_del	*^b^*Pvmit-2_C_del	*^c^*PveIF4E^1,3,4^ _PveIF4E^2^	*^c^*Pvbzip1_ A_C	*^b^*Pvmit1_ T_G
					
Line	Host group	PG-IV US-4	PG-VII US-6	PG-III NL-8	PG-VI NL-3	Proposed resistance genotype	Pv02: 48,908,259	Pv03: 4,203,361	Pv11: 9,272,542 - 9,262,459	Pv11: 9,278,765	Pv06: 27,204,768	Pv05: 36,114,516	Pv05: 36,225,550
							
							*I gene*	*bc-1*	*bc-2^[UI^* ^111]^	*bc-2* ^[*Robust*]^	*bc-3*	*bc-u* ^d^	*bc-4*
Duebelle Witte	1	***^a^***M	M	M	M	none	–	–	–	–	–	–	–
SGR	1	M	M	M	M	*bc-1*	–	+	–	–	–	–	–
^d^OV3-7	1	M	M	M	M	*bc-2*^[UI^ ^111]^	–	–	+	–	–	–	–
^d^OV3-87	1	M	M	dM	M	*bc-u^d^*	–	–	–	–	–	+	–
Immuna	2	M	M	NS	M	*bc-1*	–	+	–	–	–	–	–
^d^OV3-88	2	M	M	NS	M	*bc-1/bc-2*^[UI^ ^111]^	–	+	+	–	–	–	–
RGB	3	M	M	NS	M	*bc-1/bc-?*	–	+	–	–	–	–	–
Olathe	3	M	M	NS	mM	*bc-1/bc-u^d^*	–	+	–	–	–	+	–
Sanilac	4	NS	NS	M	M	*bc-2* ^[Robust]^ */bc-4*	–	–	–	+	–	–	+
UI-111 (DDP-077)	4	NS	NS	M	M	*bc-2*^[UI^ ^111]^*/bc-4*	–	–	+	–	–	–	+
UI-114-8	5	NS	NS	NS	M	*bc-1/bc-2*^[UI^ ^111]^/*bc-4*	–	+	+	–	–	–	+
Monroe	6	NS	M	NS	NS	*bc-1/bc-u^d^/bc-2* ^[Robust]^	–	+	–	+	–	+	–
Othello (DDP-109)	6	NS	M	NS	NS	*bc-1/bc-u^d^/bc-2*^[UI^ ^111]^	–	+	+	–	–	+	–
^d^OV3-52	6	NS	M	NS	NS	*bc-u^d^/bc-2*^[UI^ ^111]^	–	–	+	–	–	+	–
IVT-7214	7	NS	NS	NS	NS	*bc-2?/bc-3/bc-4*	–	–	–	–	+	–	+
Widusa	8	NS	NS	TN	TN	*I*	+	–	–	–	–	–	–
^d^OV3-31	8	NS	NS	TN	TN	*I/bc-2*^[UI^ ^111]^	+	–	+	–	–	–	–
^d^OV3-13	8	NS	NS	dTN	TN	*I/bc-u^d^*	+	–	–	–	–	+	–
UI-129	X	NS	NS	NS	M	*bc-1/bc-2*^[UI^ ^111]^*/bc-4/bc-?*	–	+	+	–	–	–	+
Jubila	9a	TN	NS	VN	VN+	*I/bc-1*	+	+	–	–	–	–	–
Topcrop	9b	NS	NS	VN	TN	*I/bc-1*	+	+	–	–	–	–	–
^d^OV3-32	9b	NS	NS	VN	TN	*I/bc-1/bc-2*^[UI^ ^111]^	+	+	+	–	–	–	–
Amanda	10	NS	NS	VN	VN	*I/bc-1/bc-?*	+	+	–	–	–	–	–
Beryl (DDP-055)	10	NS	NS	VN	VN	*I/bc-1/bc-u^d^*	+	+	–	–	–	+	–
92US-1006 (DDP-108)	11	NS	NS	NLL	NLL	*I/bc-2*^[UI^ ^111]^/*bc-u^d^*	+	–	+	–	–	+	–
IVT-7223	11	NS	NS	NLL	NLL	*I/bc-2*^[UI^ ^111]^/*bc-u^d^*	+	–	+	–	–	+	–
Quincy (DDP-103)	11	NS	NS	NLL	NLL	*I/bc-1/bc-2*^[UI^ ^111]^*/bc-u^d^*	+	+	+	–	–	+	–
Raven	12	NS	NS	NS	NS	*I/bc-3*	+	–	–	–	+	–	–
TARS-VR-8S	12	NS	NS	NS	NS	*I/bc-3/bc-u^d^*	+	–	–	–	+	+	–
USCR-8	12	NS	NS	NS	NS	*bc-1/bc-3*	–	+	–	–	+	–	–

*^a^NS, no symptoms; VN, vein necrosis; VN^+^, vein necrosis on upper trifoliolate leaves; NLL, local necrotic lesions; TN, top necrosis; dTN, delayed top necrosis; M, mosaic; mM, mild mosaic; dM, delayed mosaic.*

*^b^SNP markers developed in this study.*

*^c^SNP markers designed by Soler-Garzón et al. (2021).*

*^d^Othello/VAX 3 (OV3) RILs with new unrepresented genotypes for specific host groups.*

### Host Group Assays

Differential genotypes were distributed across 12 HGs for BCMV/BCMNV, and one unassigned genotype “UI 129” was assayed for the *I*, *bc-1*, *bc-3*, *bc-u*^d^, *bc-2*^[UI^ ^111]^, *bc-2*^[Robust]^, and *bc-4* markers (Table 3). Only genotypes in HG-4 and HG-5, previously reported to possess *bc-2*, possessed the *bc-4* gene. In addition, HG-4 (NS to US-6 and M to NL-3) had a mix of genotypes with the two distinct *bc-2* mutations, namely, Michelite-62 and Sanilac with *bc-2*^[Robust]^, and “UI 111” and “UI 34” with *bc-2*^[UI^ ^111]^. Moreover, all HG-4 genotypes exhibited NS to US-6 and M to NL-3 strains, regardless of the mutation.

The HG-5 genotype, “UI 114,” possesses *bc-1*, *bc-2*^[UI^ ^111]^, and *bc-4*. “UI 114,” similar to HG-4 genotypes, exhibits NS to US-6 and M to NL-3; however, it also possesses resistance to NL-8 strain, whereas HG-4 genotypes are susceptible. Soler-Garzón et al. (2021) showed that *bc-1* alone provides protection to NL-8, and the addition of this gene in HG-5 lines explains the differential reaction between HG-4 and HG-5 genotypes to NL-8. Unassigned genotype “UI 129” also carries *bc-1*, *bc-2*^[UI^ ^111]^, and *bc-4* like HG-5, but unlike HG-5 lines, it exhibits resistance to PG-V strains. Therefore, the hypothesis that “UI 129” has another unknown gene that contributes to its resistance against PG-V strains should be tested.

The HG-6 genotypes (*bc-2* and *bc-u*^d^) exhibit resistance to all BCMV and BCMNV PGs except PG-VII with BCMV strains such as NL-4 and US-6 (Drijfhout, 1978). However, most HG-6 genotypes possess *bc-1*, in addition to *bc-2*^[UI^ ^111]^ and *bc-u*^d^ (e.g., Othello, UI-31, and UI-35). Furthermore, Monroe with *bc-2*^[Robust]^, *bc-1*, and *bc-u*^d^ is also classified as an HG-6 differential cultivar (Miklas et al., 2000), which indicates that the *bc-2*^[Robust]^ and *bc-2*^[UI^ ^111]^ mutations react similarly with *bc-u*^d^. Finally, the OV3 RILs with *bc-2* and *bc-u*^d^ but without *bc-1* were resistant to PG-III, IV, VI, and VII strains in our screenings, indicating that the combination of *bc-2* and *bc-u*^d^ conditions resistance to PG-III and PG-V strains in the absence of *bc-1*.

The HG-7 cultivar IVT-7214 possessed the *bc-3* and *bc-4* genes, based on the marker assays, indicating that *bc-4*, not *bc-u*^d^ as previously considered, may be interacting with *bc-3* to confer broad resistance against all PGs, except for the newly discovered PG-VIII (Feng et al., 2015). Therefore, we are in the process of developing a differential cultivar with *bc-3* and *bc-u*^d^ to test whether *bc-u*^d^ may help to extend the range of effectiveness for *bc-3* to condition resistance to this new PG-VIII as well. However, we did not observe our *bc-2* gene-linked markers in IVT-7214, whereas its presence was reported by Drijfhout (1978). Perhaps IVT-7214 is heterogeneous, and the line we happened to test was absent *bc-2*. This is the best explanation because the results for the “Michelite-62 (*bc-2/bc-4*) *x* IVT-7214 (*bc-2*/*bc-3*/*bc-4*)” F_2_ population from the study by Drijfhout (1978) support the segregation of a single gene, i.e., *bc-3*, with *bc-2* and *bc-4* genes present in both parents.

For the HGs 8–12 with *I* gene, *bc-4* was absent in all, and *bc-2* was only detected in HG-11. The HG-11 lines all possessed *I*, *bc-2*^[UI^ ^111]^, and *bc-u^d^* genes. As was reported by Drijfhout (1978) and validated by Soler-Garzón et al. (2021), the *bc-u*^d^ and *bc-2*^[UI^ ^111]^ gene combination is needed to condition NLL to BCMNV (PG-III and PG-VI) in the presence of the *I* gene. OV3 RILs with *I*, *bc-1*, *bc-2*^[UI^ ^111]^, and *bc-u*^d^ also expressed NLL to BCMNV, indicating no additional phenotypic effect from the presence of *bc-1*.

### Allelism Tests in F_2_ Populations and F_2:3_ Families

#### Fixed Genotypes

The observed genotypic and phenotypic segregation ratios for BCMV and BCMNV reactions for each F_2_ population and F_2:3_ family are summarized in Supplementary Tables 9, 10, respectively. The resistant gene-linked SNP and InDel markers for *I*, *bc-1*, *bc-u*^d^, *bc-2* (both mutations), and *bc-4* in F_2_ and F_2:3_ families, in most cases, fit 1:2:1 (*df* = 2.0, *p* ≤ 0.05: 5.99) segregation ratios, as expected for codominant markers.

As expected, F_2_ individuals homozygous for gene-linked markers bred true in the F_3_ generation. The true-breeding F_2:3_ families for one or more markers matched the phenotypes of the parents and other DDP lines with the same marker genotypes (Supplementary Table 9). F_2:3_ families with *bc-2*^[UI^ ^111]^ or *bc-2*^[Robust]^ + *bc-u*^d^ fixed exhibited M to US-6 and NS to NL-3. Conversely, families with *bc*-2^[UI^ ^111]^ or *bc-2*^[Robust]^ + *bc-4* fixed exhibited NS to US-6 and M to NL-3. Finally, families with *I* + *bc-u^d^* + *bc-2*^[UI^ ^111]^ or *bc-2*^[Robust]^ exhibited NLL reaction, whereas those with *I* + *bc-2*^[UI^ ^111]^ or *bc-2*^[Robust]^ + *bc-4* exhibited TN to NL-8 and NL-3. These results further support a single allele at the *bc-2* locus with a differential reaction to PG-VI and PG-VII strains based on which epistatic gene, *bc-u*^d^ or *bc-4*, is present.

#### Segregating Families: US-6 and NL-3 Comparison Reactions

The F_2:3_ families with *bc-2*^[UI^ ^111]^ fixed and segregating for *bc-u^d^* and *bc-4*, which are linked in repulsion, showed 1 NS to 3 M segregation ratio to US-6 and NL-3 strains. A low recombination frequency (6 of 552 plants = 1.1 cM) was observed for *bc-u*^d^ and *bc-4*, as expected for loci 111,034 bases apart. The recombinant F_2:3_ plants with *bc-2*^[UI^ ^111]^*bc-2*^[UI^ ^111]^/*bc-u*^d^*bc-u*^d^/*Bc-4bc-4* genotype exhibited M to US-6 and NS to NL-3, and plants with *bc-2*^[UI^ ^111]^*bc-2*^[UI^ ^111]^/*Bc-u^d^bc-u^d^*/*bc-4bc-4* exhibited NS to US-6 and M to NL-3. No F_2_ or F_2:3_ plants, which were homozygous recessive for both *bc-u*^d^ and *bc-4* genes, were observed.

For F_2:3_ families with *bc-1* fixed and segregating for *bc-2*^[UI^ ^111]^, *bc-u*^d^, and *bc-4*, the phenotypic ratios of 3 M to 1 NS against US-6 and 12 M to 3 mM to 1 NS against NL-3 were observed. Thus, unlike *bc-1* and *bc-u*^d^, which interact to confer partial mM reaction to NL-3, the *bc-1* and *bc-4* combination does not exhibit partial resistance to NL-3. Again, no F_2_ or F_2:3_ plants, which were homozygous recessive for both *bc-u*^d^ and *bc-4* genes, were observed.

The F_2_ population “Michelite-62 *x* Poncho” segregating for *bc-2*^[Robust]^, *bc-u*^d^, and *bc-4* exhibited 15 M to 1 NS segregation ratios to US-6 and NL-3. In this case, F_2_ plants with *Bc-u*^d^*Bc-u*^d^/*bc-4bc-4*/*bc-2*^[Robust]^*bc-2*^[Robust]^ genotype had NS to US-6 and M to NL-3, and plants with *bc-u*^d^*bc-u*^d^/*Bc-4Bc-4*/*bc-2*^[Robust]^*bc-2*^[Robust]^ were M to US-6 and NS to NL-3. These results further support that the two *bc-2* mutations, namely, *bc-2*^[UI^ ^111]^ and *bc-2*^[Robust]^, have the same genetic and phenotypic effects.

The F_2:3_ families, with *I* gene and *bc-1* fixed and *bc-u*^d^ and *bc-4* segregating, exhibited a 1 TN to 2 dTN to 1 VN segregation ratio to NL-3, where plants with *II/bc-1bc-1/Bc-u*^d^*Bc-u*^d^*/bc-4bc-4* genotype showed TN, and plants heterozygous for *bc-u*^d^ and *bc-4* (*II/bc-1bc-1/Bc-u*^d^*bc-u*^d^*/Bc-4bc-4*) exhibited dTN reaction 2 wpi. Plants with *II/bc-1bc-1/bc-u*^d^*bc-u*^d^*/Bc-4Bc-4* genotype, as was reported by Soler-Garzón et al. (2021), exhibited VN. Additionally, F_2:3_ families with *I* gene and *bc-2*^[UI^ ^111]^ fixed and segregating for *bc-u*^d^ showed a 3 TN to 1 NLL segregation ratio to NL-3. The F_2_ population “Beryl *x* Sanilac” segregating for *I*, *bc-1*, *bc-2*^[Robust]^, *bc-u^d^*, and *bc-4* exhibited six distinct phenotypes to NL-3, revealing one plant genotype *Ii*/*bc-2*^[Robust]^*bc-2*^[Robust]^/*bc-u*^d^*bc-u*^d^ with NLL reaction, and one plant genotype *II*/*Bc-1bc-1*/*bc-2*^[Robust]^*bc-2*^[Robust]^/*bc-4bc-4* with TN. Therefore, both genotypes provide evidence that *bc-2*^[Robust]^ behaves similarly as *bc-2*^[UI^ ^111]^ in the presence of *I* and *bc-u*^d^ or *bc-4* genes.

#### Segregating Families: US-4 Reactions

The F_2_ population “Sanilac *x* Poncho” exhibited a 14 (M or dM) to 2 (NS) segregation ratio against US-4 with *bc-2*^[Robust]^*bc-2*^[Robust]^*/Bc-u*^d^*Bc-u*^d^*/bc-4bc-4* and *bc-2*^[Robust]^*bc-2*^[Robust]^*/bc-u*^d^*bc-u*^d^/*Bc-4Bc-4* genotypes exhibiting NS. Furthermore, F_2:3_ plants from “NW-63 *x* Gloria,” i.e., “NW-63 *x* UI 114” and “Gemini *x* UI-126” crosses with *bc-2*^[UI^ ^111]^*bc-2*^[UI^ ^111]^*/bc-u*^d^*bc-u*^d^*/Bc-4Bc-4* or *bc-2*^[UI^ ^111]^*bc-2*^[UI^ ^111]^*/Bc-u*^d^*Bc-u*^d^*/bc-4bc-4* genotypes, exhibited no symptoms to US-4, regardless of the allelic state for *bc-1.*

#### Segregating Families: NL-8 Reactions

The F_2:3_ families fixed for the *I* and *bc-1* genes (*II/bc-1bc-1*) all exhibited VN to NL-8, regardless of the allelic state for *bc-u*^d^ or *bc-4*. When the *I* and *bc-u*^d^ genes were fixed and *bc-2*^[UI^ ^111]^ was segregating, a 3 dTN to 1 NLL phenotypic ratio was observed. When both *bc-2*^[UI^ ^111]^ and *bc-u*^d^ were segregating and *I* gene fixed, a 1 TN to 2 dTN to 1 NLL segregation ratio was observed, which fit with the segregation ratios observed above for each gene. Thus, the *bc-1* allele exhibited dominant inheritance in the presence of the *I* gene, as F_2:3_ individuals with *II/Bc-1bc-1* genotype exhibited VN to NL-8, as reported by Soler-Garzón et al. (2021). Conversely, genotypes with the *I* and *bc-2*^[UI^ ^111]^ genes fixed, but absent *bc-1 or bc-u*^d^, exhibited TN.

## Discussion

In this study, we reported on the genomic characterization of the recessive *bc-2* gene and its genetic interactions with other genes to confer resistance to BCMV and BCMNV. GWAS with the DDP was used to locate *bc-2* to Pv11. Haplotyping in the OV3 RIL population was then used to narrow the *bc-2* region. The Vps4 AAA+ ATPase ESCRT gene, *Phvul.011G092700*, was identified, within the narrowed interval, as the candidate gene for *bc-2*. Another GWAS with the DDP combined with BLASTp of the Phvul.011G092700 was used to discover the new recessive *bc-4* gene on Pv05. The *bc-4* candidate gene, *Phvul.005G125100*, is also a Vps4 AAA+ ATPase ESCRT protein with 95% identity to the *bc-2* candidate gene.

Numerous polymorphic variants were observed for the two candidate genes. Two polymorphic variants, resulting in the translation of incomplete proteins for Phvul.011G092700, *in silico*, had different evolutionary origins. A 10-kb deletion eliminated the 3′ exons in race Durango lines, such as the “Common Red” landrace, which we believed is the progenitor for this variant. A single “missense” SNP deletion, in the first exon, was found in Robust navy bean, a landrace selection from the Michigan State University bean program released in 1915 (Kelly, 2010). The variant is also found in Michelite-62 navy with Robust as a progenitor, released in 1938, Monroe navy from “Michelite-62 *x* UI-1” (a selection from great northern landrace) released in 1953, and Sanilac navy bean released in 1956. Dr. Bill Dean, a bean breeder for many years, postulated that the *bc-2* and *bc-2*^2^ genes (now just *bc-2*) were hidden in “Common Red” and did not surface until crosses were made with great northern landrace selections, which possessed the *bc-1*^2^ (now *bc-1*) and *bc-u* (now *bc-u*^d^) genes (Dean, 1994). Dr. Bill Dean was absolutely correct in his assumption.

A single missense SNP S05_36225550 in the first exon of Phvul.005G125100 was the variant identified by comparing ORF sequences across genotypes as the most likely causal mutation for *bc-4*. Furthermore, this same variant was the peak SNP for the *bc-4* interval identified by GWAS.

The Tm-shift assay markers for the two InDel variants named *bc-2*^[UI^ ^111]^ for the 10-kb deletion of race Durango origin and *bc-2*^[Robust]^ for the SNP deletion of navy bean origin, as well as missense SNP for *bc-4*, were developed to track the *bc-2* and *bc-4* genotypes underlying phenotypic segregations in F_2_ and F_2:3_ families and for genotyping lines in the DDP, OV3 population, and HGs. In addition, the same populations were assayed for the *I*, *bc-1*, and *bc-u*^d^ gene-linked markers developed by Soler-Garzón et al. (2021). Across populations, the different variants for *bc-2*^[UI^ ^111]^ and *bc-2*^[Robust]^ did not exhibit differential reactions against the PGs (i.e., III, IV, VI, and VII) tested in this study. Thus, we bracketed the superscripts “UI 111” and “Robust” for the different *bc-2* gene mutations with the same effect to distinguish them from alleles with different genetic effects that are normally noted by unbracketed superscripts.

The *bc-2* and *bc-4* genes exhibited recessive inheritance across the segregating populations. Across all populations (i.e., F_2_, F_2:3_, DDP, OV3, and HGs), *bc-2* required the presence of either *bc-4* or *bc-u*^d^ for expression. Drijfhout (1978), and the literature since then, described two recessive resistance alleles *bc-2* and *bc-2*^2^ for the *Bc-2* locus with HG-4, HG-5, and HG-7 lines possessing *bc-2* allele and HG-6 and HG-11 lines possessing the *bc-2*^2^ allele. Conversely, we observed no alleles for *bc-2*. The sequencing data for the *bc-2* Vps4 AAA+ ATPase ESCRT candidate gene did not reveal any polymorphic variant between *bc-2* (e.g., “UI 111”) and *bc-2*^2^ (e.g., Othello) in dry bean genotypes. Consequently, *bc-2* in HG-4 and HG-5 lines interacted with *bc-4* to confer resistance to all BCMV PGs except PG-V, and *bc-2* in HG-6 and HG-11 lines interacted with *bc-u^d^* to confer resistance to both BCMV and BCMNV. *bc-2* was not observed in the HG-7 genotype IVT-7214 plant we sampled, but it did possess *bc-4*. Interestingly, IVT-7214 was susceptible to a *Clover yellow vein virus* (ClYVV) strain from Wisconsin (Larsen et al., 2008) and to BCMV “1755a” strain forming the new PG-VIII (Feng et al., 2015). Meanwhile, the USCR-8 germplasm line (Miklas and Hang, 1998), with *bc-1* and *bc-3*, was resistant to all ClYVV strains (Hart and Griffiths, 2014) and all BCMV and BCMNV strains, except NL-3 K strain to which it exhibits mild mosaic symptoms (Larsen et al., 2005). Perhaps *bc-4* negatively affects *bc-3* resistance against these potyviruses. Whether *bc-4* interacts with *bc-3* in IVT-7214 (HG-7) will require further investigation. At present, F_2_ populations with IVT-7214 as a parent are lacking in our studies because it is photoperiod sensitive and does not produce flowers in our greenhouse.

The tight linkage of *bc-4* and *bc-u*^d^ in the repulsion phase likely contributed to the study by Drijfhout (1978) describing *bc-u* as the helper gene for *bc-2* in HG-4 and HG-5 lines. In addition, *bc-4* did not exhibit any effect by itself or in combination with any other gene besides *bc-2*, whereas *bc-u*^d^ alone delays the onset of symptoms by about 1 week and interacts with multiple genes, e.g., *bc-1* and *bc-2*, to confer resistance to BCMV and BCMNV (Soler-Garzón et al., 2021).

Overall, *bc-2* helped by *bc-4* provides resistance against all BCMV PGs except PG-V, while *bc-2* helped by *bc-u*^d^ provides resistance against all BCMV and BCMNV PGs except BCMV PG-VII. This results in a major differential reaction for the *bc-2* and *bc-4* vs. *bc-2* and *bc-u*^d^ combinations, as the former is resistant to BCMV strain US-6 (PG-VII) and susceptible to BCMNV strain NL-3 (PG-VI), and the latter is susceptible to US-6 and resistant to NL-3. In addition, the *bc-2* and *bc-4* combination does not protect the *I* gene (TN, leading to plant death) against BCMNV strains, whereas the *bc-2* and *bc-u*^d^ combination does protect the *I* gene (NLL restricted to the inoculated leaf). Thus, in the absence of the *I* gene, if BCMV strains from PG-VII are prominent in a region, then the *bc-2* and the *bc-4* combination is preferable for protection. If that PG is absent, then the combination of *bc-2* and *bc-u*^d^ is the better combination to deploy.

The studies below support Vps4 AAA+ ATPase ESCRT proteins as the candidate genes for the recessive *bc-2* and *bc-4* genes, which interact to condition resistance to BCMV, which like TBSV and ZYMV, is a +ssRNA virus. The Vps4 AAA+ ATPase ESCRT candidate gene proteins for *bc-2* and *bc-4* have a key function in various activities related to endosomal traffic to lysosomes, organelle biogenesis, DNA replication, protein folding, and proteolysis (Obita et al., 2007). Barajas et al. (2014) documented a non-canonical role for Vps4 AAA+ ATPase ESCRT during *Tomato bushy stunt virus* (TBSV) replication, whereby the virus recruits the cellular ESCRT machinery for replication, evading recognition by the host defenses and preventing viral RNA destruction. For example, a study in *C. sativus* revealed Vps4 AAA+ ATPase ESCRT as a candidate gene for z*ym*, a recessive gene resistant to ZYMV (Amano et al., 2013). A critical difference between Vps4 AAA+ ATPase ESCRT-mediated resistance to ZYMV is that only one gene model encodes this protein in cucumber. In contrast, there are two such gene models in common bean, and both need to be disrupted to confer resistance to BCMV. Moreover, Feng et al. (2018) observed that the *bc-2* (and *bc-4* combination) had no effect on cell-to-cell movement and replication of BCMV but did affect systemic spread of BCMV in common bean, which fits the phenotypic reactions observed in this study and the mode of action for Vps4 AAA+ ATPase ESCRT described by Barajas et al. (2014).

The *in silico* analysis of the transcribed proteins for the *bc-2*^[UI^ ^111]^ and *bc-2*^[Robust]^ frameshift mutations showed non-functional truncated Vps4 AAA+ ATPase ESCRT proteins due to premature termination. For *bc-4*, a missense SNP mutation (S05_36225550) at codon M33R (Methionine to Arginine) in the MIT domain of the Vps4 AAA+ ATPase ESCRT protein from Phvul.005G125100 gene model was predicted by *in silico* protein analysis. Similarly, Amano et al. (2013) found variants at codons F29S (Phenylalanine to Serine) and M33I (Methionine to Isoleucine) in the MIT domain of the candidate protein for *zym* resistance gene. The latter variant is in the same codon position as our variant for *bc-4*. They suggested that the codon variants found in the MIT domain are the key elements responsible for viral-host protein-protein interactions. It is clear from the literature, as well as the results for *bc-2* and *bc-4* in this study, that host proteins that are used by the virus for replication and transport, which become non-functional or less functional due to frameshift or missense mutations, will result in recessive resistance genes in the host for combatting the virus. Candidate genes encoding altered or non-functional proteins due to missense or frameshift mutations underlie the recessive *bc-3* (Naderpour et al., 2010) and *bc-u*^d^ (Soler-Garzón et al., 2021) genes conditioning resistance to BCMV and BCMNV. The candidate gene for *bc-u*^d^ (Pvbzip1) contains a stop-gained mutation that results in a premature termination codon and non-functional protein. For eIF4E, the missense coding variants that contributed to *bc-3*, aligned with missense coding variants for eIF4E associated with virus resistance in other species, similar to the same missense codon 33 variant in the Vps4 AAA+ ATPase ESCRT candidates for *bc-4* and *zym*.

## Conclusion

The findings that were obtained herein enhance our understanding of host-virus pathogen interactions and provide new tools and information to facilitate breeding for resistance to BCMV and BCMNV in common beans.

## Data Availability Statement

The datasets presented in this study can be found in online repositories. The names of the repository/repositories and accession number(s) can be found in the article/Supplementary Material.

## Author Contributions

AS-G and PNM conceived and designed the experiments and wrote the manuscript. AS-G conducted the genomics analyses, genotyping, and phenotyping. PEM generated SNP data. All authors contributed to the article and approved the submitted version.

## Conflict of Interest

The authors declare that the research was conducted in the absence of any commercial or financial relationships that could be construed as a potential conflict of interest.

## Publisher’s Note

All claims expressed in this article are solely those of the authors and do not necessarily represent those of their affiliated organizations, or those of the publisher, the editors and the reviewers. Any product that may be evaluated in this article, or claim that may be made by its manufacturer, is not guaranteed or endorsed by the publisher.

## Footnotes

1
phytozome-next.jgi.doe.gov/
