# Supplementary material for: Coding Mutations in Vacuolar Protein-Sorting 4 AAA+ ATPase Endosomal Sorting Complexes Required for Transport Protein Homologs Underlie bc-2 and New bc-4 Gene Conferring Resistance to Bean Common Mosaic Virus in Common Bean
Source: Front Plant Sci. 2021 Dec 13;12:769247. doi: 10.3389/fpls.2021.769247 (PMC8710759; doi:10.3389/fpls.2021.769247)
Supplement: Supplementary file 1 [file Data_Sheet_1.DOCX]

Below are sequences for the bc-2 gene. The alignment of this sequences shows a gap of ~10,084 bp in ‘UI 111’ (resistant).

Additionally, these are the sequences of primers used to identify the gap.

| Pvvps4_del _Fa | AGACCGTTTGCTAGGTTCACAA |
| --- | --- |
| Pvvps4_del _R | TGTAGGCAATAAGGCGACGTTT |
| Pvvps4_del _Fb | AAATTATAAACATGTGTTGGCGAGC |

>G19833_bc-2_(this reference genome has the 10 kb segment)

AGATCCATAAATATATTTTGTATAATTTATAAATATATTCAAAATATATTAATGAATTTTTAAATTATATAATCTAAAAATATATTATAAATTATATATTTTTTAATTTTTAGATTGTATAATCCAAAGATGTATTTCAGTTTAATTTTAAGATTTTTGAATTACGTAATCTAAAATACAATGCCCAAAATTCCTTTGACATCCTTTTGCTCTTAAGTATTGATCAAACCATGTCGAGAATGGTTCGGTTCTTCCTCTTCGCCACACCATTTTGTTGAGAAGTGTACGGTGCGATTAGAAATCGCCTTATGCCTTGCTCCTCGCAATACTCCATGATAGTCGTCGAAGTATACTTGCAACCTCTATCAGATCGTACAGTTTTGATCTGTCTATCAGTTTCCTTCTCGACCATTACTTTAAACTTTTGAACACCTCGAATGCCTCAGATTTTTCTTTCAAAAAATAAACCCAAGTTTTTCGTGAGAAATCATTGATAAAGGTAATGAAATACCTTTTACGACTGAAGGATTTTGGAGTAATTGGCCCACATATGTCGGTATGAATCAATTTGAGAGGTTGCTTAGTCCAATATTGAGCCTTCTTTGGAAACAAGGTTCTCACATGCTTGCTGAGCATACATTCTTCACAAAATTTTCCCTCGTAGTCCATGTTGGGTAGCCTGTACACCATATTCTTCTTCGCTAGCTATTTTAGACCACCATGATGTAGGTGATCAAAACGGATATGCCACTGCGATGTCTTATCTTCTATGTTGACTTGCAAACATTTTTTTGGTATGCTTCTTAAATTCAGCTTGTACATTCGATTTCTTGCCATCTAGATTCGAGCAACCAAACGCCCTTACTTATTCTTCAAGTGTAGTAGCCGATCTTTTAAAAATATCGAGTAACCTTTCTCTGTGAGTTGCCCCATACTCAAAATGTTGGTCTTGAGGTATGACGTAATAAACATCTTGGAGTGACCCAATTAAGCCATCTTTTTGTAAGTAGCAAACCGTACATTGGCCTTTGACTTCCACCTTTGATGCATCTCCAAATGACACATGACCATCTTTAATCTTTTGCATCTCTTTGAATAGGTACTCGTGACCGCACATATGGTTGCTTGCTCTCGAGTCGAGGTACCACAAAGTGTCATTGTTAATGTTGACTTCATTTTGAGCCATCAAGAGAACACCTTCATTTGTTTTGTCTTCCAAGGCTAGGTTGGTTGTCTCTTCTATCTTTATATCAGATCGACAATCTTTTGCAAAATTCTCCACTTTACCACAATTATAACATTTGTCGGAGTTGCAATCCTTCGCATAGTGATCATATTTGTGACATTTGTAGCACTTGATATTGGAGTAATTTGATCGACCGCCTCTTCCTCGACCACGTCCTCTTCCACGCCAATTTGGTTGGCTCAACTGTTCCTTCTCCTTATAGTACCCTTCATGACTAGTGCCTTTACTACCATGACCATTTCCATGACTTCCACAGCCACGCCCTTTACTTCTAGAGTTTTGATGATAGAGTACTTTTTTGTCTTTGATTGACGCCTTGGTTTGAAGTGCTTACTCGAGTGTTTCCTCCTCCTTCTTCTTCTTCCGTTACTCGTGTGTCTCGAGTGAACCGACGAGCTCGTCAACAGTGAGCGTCGTTAGGTCCTTTGACTCTTCTATTGCGCACACAATACTCTCAAAATTATCGGTTAATGTTCTCAAAATCTTCTCCACAACTCGTGCATCGGTCAACGTTTCACCTTTTCGGTTGAATTGATTCACCACAGTCTGTACATGTGTAATGTAGTCAGATACACTTTACGACTCCATCATCTTCATGCTCTCCAACTCACCACGAAGAGTTTGTAGACGCACTTGCTTAACTCACTCAACTCCTTTGAACACCTTTTTAAAGATGTCCCACGCTTCTTTTGAAGTAGTTGCACTGGCAATCTTCTCAAAGCCCGACTCATCAACTGCTCAGAACAACATGTATAGTGTCGCCTTATCCTTTGATCACATCTCTTTCAACGCCTTGGTTTGAGTTGCCGTGTAACTTGCAGTATCTATTGGTTCTTCGAAACCTTCTTCGACCACCTCCCATGCATCTTGAGAACTGAGAAGAGCTTTCATTTGAATGCACCAGTTATCATATATGGTCGCCTTCGTTAACCATGGTAGCGGTATTTGACCCGTCACATTTGCCATTGGCTTTGATACCAAATTGAAAAACAGACAACCACTCACACTTGGATTTTTTTTAAAGAGAAACTCTTCTTCTTGTATTTCTCTATTGTATTTCTTTCTGTGGTGCATATTACAACTGGGAGAACATCCTATTTATAGGCTTTAGAAAGCTCTTATAGAAAAATCTTCATTTAACCTTAAAATCCCACTAATAACATAATTAAAATTTCACTCACAACTTTTGACCTTTCACCTATCCAAACTCTCGTTCTACTACTTTATAGTAACTTATAATTTTAAAACCCACAGAACATGATCATCATTTGCTGGACTCATATTAGTATCTTTCACAACAATATTATTTGCGACTACTACAAATTATGTTTACAGTGCACAAGTTTCATTTCAATGTTCTTTTTCTTTTAAAACAAAAGCTATGGTGCTGGAAAAAAGTAAAATCAAGCTAAGTATTAATTGAATTTACTGATGTCGACTTTCAGGATTCAAGAAGTACAAAACGGTGATCTCAAAGAAAGCAACAAACTAAGATTCCAAAGCACAGATAATGGTTTCGATGAAGTCAATCAAAGGAACCAAAGAAGATTCAACACTAAGAAGTTCTTCAAGATAAGGCTTGATAATTCTTCCAATTATCTAGATTTTTAGTTTCCTTTTAGATAAAATATGCTTTTCTTCTAAATTATTCCTTAAGAGTGTCCACTTTATTACAGTAAAAGAAATTTGTTTGATTAAAAACTTGTGTTCACTTTGGATTATATTCATTTTAACTTAAATCTTTATCCTTTGTTTTCGGGTAATAAAGAGGAGAAAAATTTAACCAAAATGGAAAATTGAAAATCACAATAACAGTTTAAAAACGTCAAGAACACAGCTTGGAGAAATATTCAGTGTACTTTGGAACTGTTTCTTCTCATGTGTCTTCTTTGAGAGACAATCATGTCAGAAGACATTGGTCGAACATGTAAACAACCCTTCCTGCAAGAAATTGAACCATAGTTCACAACATTGGAGCTTGTACTTCATGTGATATTCAGCTATTAAAAGGTGCTTTACATATTTTCTTATTCAAAGTATTGTTATTAATTCAAGTAAGATTTGATTTCACTTCCATACCAAATTTAGAAAAAGAAAAACGGACAATGTTATTAACATATTTAGTTGTTCTGTTTTAAGTTATTGAACTTTGTTTGCTTGACCAAACTTTCATTTTCTGTTTCAGCTAGGTCTTAATCTTTTTCTTATATCACTATGTATTTTATTCTCTAATACACAAGTTGGGGTGTGTGTTGGAGATCCCACATCGACTAGAGATAATGACATTTAATTGTATAAAAGTGGGTGCAAACCTCACCTTATAAGCCGGTTTTATAAGCCGGTTTTATGAGGCTGATTTAGTCTTAAAGTTTACTTCTTAATAAATGAAACAATCAATTTTATTTGTAATTATGGTAAGGCAATATGCTTATTGCTTCACGTTGATGATAATTGTTACATTACAGGTATAATGGCCTACACTAACCTTTCAAAAACTAGACAAAGTTTAACCACAACAAACACATTGTGCTTACATAAAAATCACCTGGCTTTGACAAAGGTTAACCATCCAGAAAAAACACAATGATACAGTGGCAATACAATACTAATTATTTTTTCCATGATTCAGAGAACATTTGCACTGATGATCACAGGCACCTGACAATATTTGTTTGAGTTGAACCTTTCTTTTATTGCAGAAATGAAAGTGATATAAATAAATGCATTTTCTTAGGTGTAAACTCAATAATGGTAAGGACCAAGAAGCACTCCACATTAAGTAGAGAGTACATTGAGACCAATAGGACTCCTGACCCTATGTTTGCCATTATTCCATATCAAGGAGCCAAACACAAAGTTTCCATTGCTTTGCTTGAAAGGGAAGAAATCAATCCTGAAAGTTATCTTCTCCCCAGGTTTCCCGAACTTCAATTCTGCTGGAGTAACTGTAACCTTCACACCAGATGGATTTTCAATACTTGCAGAATACACTGTTGGCTCCTGACCATAATAAGTAACTGTACGATAAACTGATAAACTTCCATTCAAGTTGGACACACCAATTGAAGGATAATTGAAGTTGTTGGAAGCTGTAAGAGGTTTATGGCATTGAGTAAGATCCCCAGTGAGGTTTTTAAGTTGTGCTGGGCTTGCTCCATTGCTGCACAGAAAATTGAGAACATCTTGGGAATGAAATTCATATACTAATCCCGGATTGAGTGATGCAACTGGGTTAACATGTCCAGATCCATAGTCAAATGGTGTGGTTTGAGTTCCATTTGGATCTCGTCTTATGAGGTGGTGTGTGTTATCCGTAACTGTTGCTGTAACATTGGGAAAAAACGGTGATCACAATAATAAACATCTATTCTCATGACTTTGGTAGTTGCCGGGAACTTTTGAAGCATGAAAAACTTACCTGTTGTCATTATTGAAGACATTATGGCTGCAGGACCCCAAGATGGGTGGTGAGATTTTATAATTGCTGCAACTGCAGTTATGTGTGGGCAAGACATTGATGTTCCTGAAATGATGTTATAGTTGACAGGTCGTTGTTCAACTGTGGCATCAGTTCCTACTGGAGACCATGCTGCCAAAATATTCACTCCAGGTCCTGTGATATCTGGCTGCAGAGTTGGATTATAGCACACTATAGTTATCATACATGAGTATCTATGTCCTCTATATTCTTTCAGTGTCAACAAAAAAAGGTTAACTAGGAAGAATCCAGTCCATGCAGTCCAAACATATAGTCCAATGAGTCTAAGGAAACTTGCCTTAATAATGTCTGGTGTTATTATATTTGGCCCTATGGAAGAAAAAGCTGCCATTTCTGGTGCTGGTTTGGTACCAACTACAGTTACTGTTGGGATGATTCTTGCAGTGGGATTCCTGACATAATAAAGCATTAGTGTACGATGTTTTATGGATCAATCTTCTGACAATGAAGTGTGACTAACTTTGATATTAGAGATTTGTTTCTCTATTTGACAAACTTTTAAAACTCACTTATCTGTCTTTACATATGCTTGAAGCTCTTCTACAGCATCCTGACCAATGAGAGTGCTTGGGATGACAAATTGAAAACCAATATCTTTGGCATTATGATCAATAAGTATCATTCCAACACCCCCACCTTGCCTTATTTGTATAGCCTTCTCTTGTCTATCATCACTGAAATTCTCGATTGTACAGATCACAATTTTACCCTTGATTAAGGTAGGATTCAGAGTATTGTTCTTGCAGAAGCTGTTACATGGAGAAATCAAGGAAATAGGATTAAAAATTCAATCATGTCACTCTAGATGTCTTGGACTTAAAAACATTTAGCAACAACAAAGTTTGTGCTTAGCCAAATAACAAAGAGAATACCTTGCATTTATAGCTGGAACTCCAGCAGCTGCAGCAGCACTTCCATATATCAAACCATGCGAGTGCTTCATTTTTATTGGATTCAAAGAAGAACCCTGATGGCATTTATGAACCATTCTTAATTTCGTAACTAAGGTTGATATGCATTAGATGTATATTAGTGTTAAAATTTAGAAAATAGTTCGTTATTGACTTCACCTTTAAGACCTTTGAGTTACCAAGGTAGATATTTGAACTGAATTCCCTGTCTATAGAGCTAGCAGCAACAGTGAGGATCCAAGGAGCAACATTGCAGGCAGTACGTGGAAAAACTGAGTTTCCAGCTGAAGCAGAAACAAGAATACCCTTTTGAAATGCATGGAATGTTCCTAAACTGACTGCATCTTCGAAGTAAATTGGCTGGGGAGGATTAGGACCAAGAGAAAGAGATAGTATGTCAACGCCGTCATGGATAGCATCATCCACTGCTGAAAGAATATCAGCATCACTGCAAAACCCAAACCAACAGGCCTTGTAGATGGAAAGTCTAGCACTTGGGGCACCACCTCTAGCTGTTCCTTTGGCAATGCCAAATAAGCTGGCATTAGCAACAGTGGACCCTGCTATTGTGGAAGCTGTGTGTGTTCCATGTCCATCACTATCTCGAGCTGACCGGAAAAATATCTTACTGCCAAAACCGTCTAGAGGACCATTTTCTGCTTCAAACCCTTTTGAATAGAACCGAGCACCAATAATTTTCCTGAAAAATCCAAACTCCAGGTGAGTGACATAGTTATCAAATGACAATCTCTACATATCTATAGCATAGCACAAGTTTCTGCCATAAACCTGCTATCGAAAAAGTTTAGCCTATGGGGTGTGGGAACATGAATACTTGTATGATGAATCTGAACACATTCATTCATGTAAGAGAATGCTATGAACTTTGAAAGCGTGGATAATATGGATAATACACTGGTCCATCTACCTTGTGCTGAAATCTAGTTTTTCTTATTAGAAGAATTAGGTTGGCAAAGTTAGAAGTAGAAATCTAGACCACTTTGTCATAGACATCCTAATAGCGTGTCACATATTTTAAACTATTAGGTGAAAACATGTGACTGATTTTACATTAAGCTTTAACAGCTTCAAATGCATAATTTATTTACTTCAATATAGTAATCCATCTTAAAAGCAGTCTATGATGGATTTTGAATTCTTACTTGTTGCAATTGGCTAGTGTAAACTTATCACCAGCAACACACTCTCCTTTGAATTTTTTGGGCACGGGACCTACTCCATGATCGGTGAAGCTTTCCGATTCTGGCCAGATTCCTGAAAACCAATGTTGTTGAGTCACTCATTTTTAATCAAAATGACAGAAAAAAGGAGTGTAAAAAGAAAGTATTAACCAGAGTCAATGACGCCAACAATGACTTCGGATGCGGTGTCTAATGCTTTAGGATTGCTCAAGTAGATAGTATCCAATCCAAGAAAATCCCAAGAATGTGTTGTGTGGAGTTTATTCATTTTGCTCTCAAAAACGGACACTACAGATTTATGTTCTGAAACACAATTCAAAAACGTTGTTCAGAGCCTGAGCAAGTAAATGGTTGGTGGATTCCATATAGAATATGTTTTTTCACTCTGAAACTTGAGCAAATACAACTCCAAATTGGAGCCACATATTTTAAATTGAGAAGCCATTTTCAAATAGTGAATTAGTCCTCATTGTTTCCTTGTCCAATATAGTGGAAAGATGAAGACAGAAGCATATAACACTTGAATACAATACCTGCAAGTTGATGAGCTTGCTCAGGTGTAATCATAGCTGAGAAGCCTTGAAAGCTTTTAGAGTAATGGTGGATGGTTGTTGCCTTTGCTTCACTCAGACTGAAAAGTAGAAAGAAATGTACACAGGAAGTCCATTTTGGCCAAAATTAGTCCTTTTCCAAATAAATTAAAATTAGAAGAACACTGATAGTTAGAATTAATTCCATGAATTCTATCTCAAATTGGGTGACATGCCTTCCAGTAACTGAAGCTAATATCTCATGGTTTGCTCTGATTACGGATTCTGAATTAGGGTGTGAATGATCTCCCATATAAATTATATGATGCTGTATCAAAGCAAATCCAAGAGTTAGTTTAGAGCTTCTATTGTAGCTCAGAGGCTGTGCATGCATGCAACTCTCGTAGACACCATGCATGCATCAAGTCCAAGTGAATATGCTCCTTACCTTCGGGGTGGATCCATTTACCAAAGTATACCAAATGAAAAGGAGAAGGGAGGAATAGCCTAAAACTTTGGTAAACCCCATGATACACTGAAAGGGTCCAGAATAGAAAGCTGAATGTGAAAATGGTGACTAATTTTATGGTTGGTGATTCGTCCATTTTATAGCCAAAACTGACAAACTCACTTGCCCTGACATATAAAAAATTCAGTTGCCGGCTTTATATTTTTCTGTTTATTACTTGGTGGGATTCTTTAGAAAGTAAAATGACATTCACAAAAGGAGATAAGATGAATATATATATATATATATATATATATATATATATTTAACGAACTAACAGCATTTGGACGAATTATAAACAGCAATTATTGGATTCAAACTTGTATCATTCACAGCTGCATTGTAGAGAACTAACACAAATTGTCCAATATTTAAAACATAAAAGGACGATGGGTCTTCCATTTGTCTGAATTTGAGAGTGTTATTAGCAACCACAAATGGCATATGCTCACCCAGATACCAAAAGCTACAGTACAATAGAGTCTTTTAGAAAGAAAAAAAAACAAACTATAGTTCTTTACTGCATGCTTCTTCCAAAGATACTCCGTTATTCAATTGTTTCTGCTATAGGTGTTTATGAGTTAGATCAGATTTTGAAAAAAAAGAATCCAGAACAATCTATTTTCATTGGTTCAGATTGGTTTGATTTTTCAAAAAAAAAAATGCAAAATCCAAATCAAACCGGTTCAATAAAAAAAGAATCGGTTTAATTTGAAATAATTCAATCATATATGTAAATACACAATTTTGTCTTAAGATAACTACAATTTTGTCAAAAAAACTCAGAGTTCTATCATTAAGAAAAATAAACTACTTTTATAAGTTAAATAATAAGAAAAATTATAAAATTCATATATTCATATCAGAAAATAGTTAATAGTAAAAAAAATGCAGCACTAAGAATCCGAACAAACGTGTCCAGATTTACGTTGGCATACCAAGTAGAAATGATTGAATCCTTCTACTGAGGTTTTCTTGCTTAACACTAGTTTTTCATCTGCATCACAAACTATTTTGAATTTATATGTCCATTTGGCTGATCACCTCCCTCACACTTAGAACTGATCGTAACCTGACAGGTTACACGTTACTTCAAATCATCATGTGATTGAAACAAAGAGGTGACTGAAAAGTTATCTGTTTCTGTTGTTGACTTCTAACTCTATCAAGAAACCTTGCCAAGAATGTTTTCACATAGGTGAATCACACCTTATACAATTTACATGCTCTTTTAGGAGTAATTTGCAGTGTAAACTAATCTCACATCTGGTTGAACAATCTGTTGAAAACTATATTTTATGGTTAAATTAGTCTGTCTGTTACAGCTTGGTGAAGCACAGCAGCTGAAATTCCCAACTCCCCTAGAATTTGAAAAAAACACTCAAACCTAAAAAGAATCCTACATTGAAATGCAGACAAACATGAGTAACCATCACCACATATTCACATGAAATTTATTTTTCTGATAATTTCAGACTGTTTTTATACACAATTTATGAGAAGTAAAATTATGCATTTCACTTGATCCAATAGTTCTGTAAATTACAAATCATAACATGTTTAAACTAGTGCAGATAGTCGAAGAAAAATTCCGTCAGAATTATCCTTCCTCTCCGAACTCCTTTGTGAATCTCTCATGAACATCGAGGTCGGCCTTGCTTACAGTGGGTCTTTGTCTAGCAAGTACTTTGTCAAAATCTGTCCTCGATATAGGTGGTGGCAGGATCTGTAGAGCAAACAGGAACATAAAAATTATTACTCTTCAATAATGAGTTAGAAAATGAAATTTTGGAGTCCTACTGTGATCAAAATGTGCATCAGCTTGAATACCTGTGAAGCAAGTCCTTTCGCAGCAAGCTCCTGCATTGTAATTTGTATTGCACTTTGTTGCTTTGCTCCACATGGAATCCACATACCCTCAGAATTCTTAAAGAAAAACATGGCATCTTGGGTTTTGCGAACAGGTTCAAATAAAACATCTTTCACCTGTAGAAAAATTACCTTTACCTTGAAATTCATCTAAATAAAAATTATCATTGTAACCAGGTATTTCACATAGAAGTCCAACTTACACAGACAGATATATCTGAACCTGAAAACCCCCCTGTCTTGCGAGCCAAATGTTCAAAATCGCTTTCGGTCAAATTATGGGGAGTATCTCCTAGGTGAACCTGTTCAATTTATCAACCAAGAAAAAGGCATAGTAAGACCGTTTGCTAGGTTCACAATACATAATAAGACCAATCCTTCGATGAACATCATCGTGTATAATGCCCATACCTTGAACATGTGTTGGCGAGCCTTCAAATCTGGTAGGGGTATGTATATTCGCTTATCAAAACGTCGCCTTATTGCCTACAATAACAACTCGATTAATCAAAATAGTATCCCGTTGACTCAGAAAAGAATTAGTAACCAGAACTATTTTGTTCTTACCTGGTCTAGAGCATAAGGTGTATTTGTAGCTGCTAGAACAAGAACCTTCTGATCATTGTGTCCTACACCCTACAACGTAAATAAAGTGGAAAACCTACATTATGATGGAAACCTTGAATCTTCTGAATTAGTACAATAAGGGTGCATAATAACATTAATTACAGGGGGAGGGGATAGTTCGAACACTACTTTTTGAAAAAAAAGATACTAGCTACTGTGTTCAGCCTAATAAAAAAAAGCTAAATATGACAAAGACTCGAGTCATTAATCAAAAAGTGCGTGCAACTTTCTATATGGCTAAAAATCATCCAATATGTTACTTTACAGATTCTTTTACAGGAAAAAAAAGGGTAGTAATACTATAACCAAATCTAGGCCTGGCCAGTCAAGTGATTATGGATGCCAAAAAAAAAAACAACTGCAAATGTTCACACGACT

>UI-111_bc-2_(this genotype lacks the 10 kb segment) AGATCCATAACTATATTTTGCATAATTTATAAATATATTCAAAATATATTTATGAATTTTTAAATTATATAATCTAAAAATATATTATAAATTATAAACATGTGTTGGCGAGCCTTCAAATCTGGTAGGGGTATGTATATTCGCTTATCAAAACGTCGCCTTATTGCCTACAATAACAAGTTGATTAATCAAAATAGTATCATCCCATTGACTCAATAAGAATTAGTAACCAGAACTATTTTCTTCTTACCTGGTCTAGAGCATAAGGTGTATTTGTAGCTGCTAGAACAAGAACCTTCTGATCATTGTGTCCTACACCCTACAACGTAAATAAAGTGGAAAACCTACATTATGATGGAAACCTTGAATCTTCTGAATTAGTACAATAAGGGTGCATAATAACATTAATTACAGGGGGAGGGGATAATTCGAACACTACTTTTTGAAAAAAAAAGATACTAGCTACTGTGTTCAGCCTAATAAAAAAAAGCTAAATATGACAAAGACTCGAGTCATTAATCAAAAAGTGTGTGCAACTTTCTATATGGCTAAAAATCATCCAATATGTTACTTTACAGATTCTTTTACAGTTAAAAAAAGGGTAGTAATACTATAACCAAATCTAGGCTAGGCCAGTCAAGTGATTATGGATGCCCAAAAAAAAAACAACTGCAAATGTTCACACGACT
